# Effects of *Cassia abbreviata* extract and stocking density on growth performance, oxidative stress and liver function of indigenous chickens

**DOI:** 10.1007/s11250-019-01979-y

**Published:** 2019-06-21

**Authors:** Martha C. Jobe, Cyprial N. Ncobela, Nokuthula W. Kunene, Andrew R. Opoku

**Affiliations:** Faculty of Science and Agriculture, University of Zululand, KwaDlangezwa, 3886 South Africa

**Keywords:** Average daily gain, Liver enzymes, Plant extract, Stocking density, Superoxide dismutase

## Abstract

The objective of the study was to investigate the effect of stocking density and extract from *Cassia abbreviata* stem bark on growth performance, oxidative stress and liver function of indigenous chickens. A total of 420 1-day-old female Ovambo chicks with initial body weight of 0.32 ± 0.036 kg (mean ± SD) were used in the study. Birds, which were cooped in stocking densities consisting 5, 10 and 20 birds/m^2^, were orally administered with 0, 50, 200 and 500 mg/kg of extract from *C. abbreviata* stem bark. Each stocking density per dosage level of extract was repeated three times. Average feed intake was lower (*P* < 0.05) in birds housed at 20 birds/m^2^. There was a low (*P* < 0.05) average daily gain in birds housed at 20 birds/m^2^. Malondialdehyde was higher (*P* < 0.05) in 20 birds/m^2^. Average daily gain was high (*P* < 0.05) in birds administered with 0 and 50 mg/kg of *C. abbreviata* stem bark extract. Birds administered with 0 and 50 mg/kg of *C. abbreviata* stem bark extract had a higher (*P* < 0.05) gain to feed ratio. Birds dosed with 500 and 200 mg/kg had high (*P* < 0.05) superoxide dismutase activity. Birds administered with 500 mg/kg of *C. abbreviata* stem bark extract had the lowest (*P* < 0.05) malondialdehyde. A 500 mg/kg of stem bark extract from *C. abbreviata* resulted to higher (*P* < 0.05) activities of aspartate transaminase and alanine transaminase. There was a significant (*P* < 0.05) interaction between the stocking density and *C. abbreviata* extract on catalase activity. High stocking density of 20 birds/m^2^ reduced growth performance and induced stress in indigenous chickens. High dosage of level 500 mg/kg of stem bark extract of *C. abbreviata* reduced oxidative stress while depressing growth performance and causing hepatotoxicity in birds. There is a need to precisely determine the maximum dosage level of *C. abbreviata* extract to improve growth performance and reduce oxidative stress and hepatotoxicity in indigenous chickens in high stocking density.

## Introduction

The commercial value of indigenous chickens has been gaining attention due to their potential to contribute to economic well-being and national food security (Ncobela and Chimonyo, 2015). This is driven by, among others, exponential increase in human population and consumer choices and preferences. Indigenous chickens are preferred due to tastier meat and their propensity to deposit lesser fat than exotic chickens (Gwala, 2014). Indigenous chickens dually provide nutritious meat and eggs (Odunitan-Wayas et al., 2016). However, the stocking density standards for indigenous chickens kept under intensive production systems are unknown. This makes it difficult to intensity their production performance without deterring their wellness. Farmers rely on personal experience in determining the space allowances and use stocking density standards for broilers as a reference point (Tong et al., 2012). This is inappropriate and may lead ethical and welfare concerns. Therefore, there is a need to determine the optimum-stocking density for indigenous chickens kept intensive production system.

Estevez (2007) defined stocking density as number of birds or the total live weight of birds per unit area. For commercial chicken strains, Madilindi et al. (2018) suggested that a stocking density of 35–40 kg BW/m^2^ is suitable for broilers in 42-day production cycle grown in tropical and subtropical environments. Indigenous chickens being hardy, small-framed and slow-growers should in principle be kept at higher stocking densities. However, Dawkins (2018) argued that it is vital to be precise about the space required by birds to perform specific behavioural activities that either can effect health or be important to the birds themselves. In light of this, the temperament of indigenous chickens such as Ovambo breed, which has not been properly documented, could dictate that they need greater space allowances. This highlights the need to determine stocking density standards for indigenous chickens without compromising their well-being.

High stocking density reduces feed intake and growth performance and negatively affects health status (Goo et al., 2018). Stress, as a result of overcrowding, results to metabolic disturbances that cause excessive production of oxygen-derived biological free radicals known as reactive oxygen species that leads to oxidative damage of biomolecules such as lipids (Droge, 2002; Yun-Zhong et al., 2002). The damage of lipids (also known as lipid peroxidation) happens when free radicals abstract electrons from unsaturated fatty acids. Simsek et al. (2009) defined lipid peroxidation, an indicative of stress, as an autocatalytic mechanism leading to oxidative destruction of cellular membrane. Authors further reported that crowding enhances malondialdehyde (MDA), a main final product of lipid peroxidation, in broilers. Such information is also relevant to indigenous chickens, particularly aggressive breed such as Ovambo chickens that were known to be hardy and less stress-sensitive. Depending on the intensity of stress, enzymatic antioxidants such as catalase (CAT) and superoxide dismutase (SOD) are involved in the mechanism to protect cells from oxidative stress (Lin et al., 2004). Aspartate aminotransferase (AST) and alanine aminotransferase (ALT) activities are indicators of hepatic health status such as possibility of cytolysis.

To simultaneously improve wellness and profit of indigenous chicken production, there is a need to valorise antioxidant-rich medicinal plants in birds kept under high stocking densities.

Various plant extracts have shown potential to reduce oxidative stress (Surai, 2016). The use of medical plants such as *Cassia abbreviata* to mitigate oxidative stress and toxicity of the liver while enhancing growth performance needs exploration. *Cassia abbreviata* is a small- to medium-sized–branched umbrella-shaped deciduous tree with distinctive cylindrically shaped fruits (Sobeh et al., 2018). This perennial tree belongs to the family of Caesalpiniaceae. It is widespread in tropical and subtropical regions such as Gabon, Swaziland, Kenya, Tanzania, South Africa, Botswana and Zimbabwe (Mongalo and Mafoko, 2013). *Cassia abbreviata* possesses anthocyanins, anthranoids, anthraquinones, polyphenols and tannins (Mongalo and Mafoko, 2013). These phytochemicals have the antioxidant effect and may have a direct bearing on wellness of birds. In our preliminary study, in vitro analyses showed that stem bark from *C. abbreviata* has the ability scavenge synthetic radicals such as 2,2-diphenyl-1-picrylhydrozyl and 2,2′-azino-bis (3-ethylbenzothiazoline-6-sulphonic acid) and biological radicals such as superoxide anion, nitric oxide and hydroxyl radicals. The role of *C. abbreviata* extract as an antioxidant source in indigenous chickens kept in different docking densities is, to our best knowledge, reported for the first time. The objective of the study was, therefore, to determine the effect of stocking density and stem bark extract from *C. abbreviata* on growth performance, oxidative stress and liver function of indigenous chickens. It was hypothesised that dosage levels of *C. abbreviata* extract counteracts poor growth performance, oxidative stress and hepatotoxicity caused by increasing stocking density.

## Methods

### Study site

The study was conducted at the University of Zululand farm, situated in the University premises at Empangeni, in the Northern KwaZulu-Natal, South Africa. The farm lies at 28.8415° S and 31.8263° E. The ethical committee of the University of Zululand approved the use and care of chickens (UZREC 171110-030 PGM 2014/124).

#### Collection, extraction and phytochemical screening of Cassia abbreviata stem bark

*Cassia abbreviata* trees and their stem barks were identified and freshly harvested at Biaba, which is located in the Limpopo Province, South Africa, and transported to the Department of Botany, University of Zululand. Stem barks were samples from different *Cassia abbreviata* tress. A voucher specimen (Ramulodi and Jobe MC/01Unizul) was kept in the Herbarium in the Botany Department. Stem barks were eventually chopped, air-dried at room temperature and grounded to powder (pass through a 2-mm sieve). The stem bark powder was extracted using maceration method. Briefly, 200 g of stem bark powder sample was macerated with methanol on an orbital shaker machine at 157 rpm for 24 h, at room temperature. The ratio of the stem bark to solvent was 1:5. The extract was filtered using Whatman filter paper. Qualitative tests were done for saponins, alkaloids, phenols, terpenoids, tannins and flavonoids for the stem bark using methods described by Harbone (1973). Phytochemicals present in the plant were determined with precipitates formation and colour changes upon the corresponding tests present (Mosa et al.*,*^2012^). Phytochemical screening of the stem bark methanol extract of *C. abbreviata* revealed presence of terpenoids, alkaloids, saponins, tannins and flavonoids. Quantitative tests for total phenolic content and total flavonoid content were performed according to Kahkonen et al. (1999) and Ordonez et al. (2006), respectively. *Cassia abbreviata* stem bark extract contained 3.82 mg/g of total polyphenolic content and 2.75 mg/g of total flavonoid content.

#### Bird management and experimental design

A total of 420 1-day-old female Ovambo chicks, with initial weight of 0.32 ± 0.036 (mean ± SD), were used in the study. Female chicks were used to avoid sexual effect variation. Chicks were hatched after incubating the eggs for 21 days in the University of Zululand poultry unit. Chicks were vaccinated against Newcastle and Gumboro diseases and were reared in the brooder house. Forty chicks were kept in each pen within the brooder. The temperature was maintained at 32 °C during the 7 days and then gradually decreased by 3 °C until a basal temperature of 22 °C was reached. A standard commercial broiler starter was given to the chickens from day 1 to day 42. From day 43 to 49, birds were moved to the floor pens with sawdust bedding within the house. Birds were given 7 days (day 43 to 49) to adapt to a standard grower diet before data collection commenced. The data collection began from day 50 to day 96. The starter and grower feed were bought from Meadow Feeds®, feed company located in Pietermaritzburg, South Africa. There are no feeding standards and requirements available specifically for Ovambo breed. Therefore, a standard diet from the Meadow feeds® for growing broiler chickens was used. Diet contained 880 g/kg dry matter, 160 g/kg DM protein, 25 g/kg DM fat, 50 g/kg fibre, 6 g/kg DM calcium, 5 g/kg DM phosphorous and 9 g/kg DM lysine_total_. The feed and water was freely and continuously provided using a tube feeders and nipple drinkers, respectively. Birds were grouped into three stocking densities, which consist of low (5 birds/m^2^), medium (10 birds/ m^2^) and high (20 birds/m^2^). Grouping of birds was done on day 43, a day when they were introduced to standard grower diet. Birds, in each stocking density, were orally administered with solution containing different dosages namely 0, 50, 200 and 500 ml/kg of stem bark extract from *C. abbreviata*. Each stocking density per dosage of *C. abbreviata* was repeated three times. A randomized factorial design was used in the study. The dosing was done once a day at 8:00 in the morning. The administration of *C. abbreviata* was performed using a gavage crop needle-feeding syringe.

#### Measurement of growth performance

Feed intake and body weights were measured weekly in the morning. Weekly feed intake was determined by weighing the feed out and feed in per week. Average daily feed intake (ADFI) for each week was calculated by dividing weekly feed intake by seven. Average daily gain (ADG) was determined by dividing the difference between body weight at the beginning and the end of each week by seven. The feed to gain ratio (F:G) was determined by diving ADFI by ADG.

#### Blood analyses

The blood samples were collected in the wing vein at the last day of the experiment using 23-gauge needle and syringe. For blood collection, six birds (2 birds in each replicate) were sampled from 5 birds/m^2^, 12 birds (4 birds in each replicate) from 10 birds/m^2^) and 15 birds (5 birds from each replicate) from high stocking density of 20 birds/m^2^. Collected blood samples coagulated at 25 °C temperature and were centrifuged for 10 min at 1000 × *g* immediately after collection. Serum was extracted and transferred into polypropylene tubes and kept at − 20 °C for preservation pending analyses. The antioxidant activities such as SOD and CAT were analysed using their respective commercial essay kits. Lipid peroxidation was also estimated based on MDA content using assay kit. The assay kits were obtained from Sigma-Aldrich®. The AST and ALT were analysed using the ultraviolet method (Bergmeyer et al., 1986).

#### Histopathology analyses

After blood collection, two chickens from each group were humanely slaughtered following the abattoir protocols to collect the liver samples for histopathology analyses. Liver samples were fixed immediately after collection into 10% neutral formalin prior to preparation and analysis. The histopathology analysis was performed at the Veterinary Diagnostic Laboratory, Pietermaritzburg, South Africa. The method used allowed for unbiased description of the histological lesions which may be present or absent in the samples.

#### Statistical analysis

Data were analysed using the statistical package for social sciences SPSS (SPSS, 2012). Effect of stocking and stem bark extract from *C. abbreviata* was determined using multivariate analysis of variance (MANOVA). Means were separated using Student-Newman-Keuls (post hoc). The values were considered significant when the probability is less 0.05*.*

The general linear model used was: *Y* = *μ* + *P*_i_ + *W*_j_ + (*P* × *W*) _ij_ + *E*_ijk_.

Where *Y*_ijk_ is the response variable (growth performance, oxidative stress and liver function); *μ* is the overall mean common to all observations; *P*_i_ is the effect of stocking density; *W*_i_ is the effect of dosage of *C. abbreviata*; (*P* × W) _ij_ is the interaction between the stocking density and dosage of *C. abbreviata*; *E*_ijk_ is the residual error.

## Results

### Effect of stocking density on growth performance, oxidative stress and liver enzymes

Average daily feed intake and average daily gain differed with stocking density (*P* < 0.05) (Table 1). Average daily feed intake was low (*P* < 0.05) in birds raised in stocking density of 20 birds/m^2^ and high in birds kept in stocking density of 5 birds/m^2^. Average daily gain was low (*P* < 0.05) in birds cooped in 20 birds/m^2^ whereas it was high in 5 birds/m^2^. Gain to feed ratio was not affected (*P* > 0.05) by stocking density. Stocking density did not (*P* > 0.05) have an impact on the activity of SOD. Malondialdehyde was higher (*P* < 0.05) in 20 birds/m^2^. Stocking density did not affect (*P* < 0.05) CAT activity. Stocking density did not influence (*P* > 0.05) activity of ALT and AST.

**Table 1:** Effect of stocking density on growth performance, oxidative stress and liver function of indigenous chickens

	Stocking density (birds/m^2^)				Significance
	5	10	20	SEM	
Growth performance					
ADFI (kg)	0.14^a^	0.10^b^	0.04^c^	0.02	**
ADG (kg)	0.04^a^	0.03^ab^	0.01^c^	0.00	**
G:F ratio	0.28	0.30	0.27	0.09	NS
Oxidative stress					
SOD (%)	62.2	54.1	53.8	1.02	NS
MDA (%)	16.5^b^	16.0^b^	19.1^a^	0.09	*
CAT (μmoles/min/ml)	0.07	0.07	0.06	0.03	NS
Liver enzymes					
AST (units/L)	3.85	3.82	3.89	0.18	NS
ALT (units /L)	34.3	33.9	34.1	3.48	NS

Values in the same row with different superscript letters differ (*P* < 0.05) *P < 0.05, **P < 0.01, *NS* not significant (*P* > 0.05)

*ADFI* average daily feed intake, *ADG* average daily gain, *G:F* gain to feed, *SOD* superoxide dismutase, *MDA* Malondialdehyde, *CAT* catalase, *AST* Aspartate aminotransferase, *ALT* alanine aminotransferase

### Effect of Cassia abbreviata extract on growth performance, oxidative stress and liver enzymes

Increasing dosage level of *C. abbreviata* did not affect (*P* < 0.05) average daily feed intake (Table 2). Average daily gain varied (*P* < 0.05) with dosage of *C. abbreviata* extract. Average daily gain was high (*P* < 0.05) in birds administered with 0 and 50 mg/kg of *C. abbreviata* stem bark extract. Gain to feed ratio differed (*P* < 0.05) with dosage of *C. abbreviata* extract. Birds administered with 0 and 50 mg/kg of *C. abbreviata* extract had higher gain to feed ratio. The dosage of *C. abbreviata* extract influenced (*P* < 0.05) SOD activity. Birds, which were administered with 500 mg/kg, had higher (*P* < 0.05) SOD activity. Dosage of *C. abbreviata* extract affected (*P* < 0.05) levels of MDA. Birds that received 500 mg/kg had the lowest MDA whereas those that had a dose of 0 mg/kg had the highest MDA levels. The activity of CAT was not affected by dosage level of stem bark extract from *C. abbreviata*. The activity of AST varied (*P* < 0.05) with dosage level of *C. abbreviata* extract. A dosage levels of 200 and 500 mg/kg of *C. abbreviata* extract had the highest (*P* < 0.05) AST activity. Birds that did not receive *C. abbreviata* extract had the lowest (*P* < 0.05) activity of ALT.

**Table 2:** Effect of dosage level on *Cassia abbreviata* extract on growth performance, oxidative stress and liver function of indigenous chickens

	*Cassia abbreviata* extract (mg/kg)					Significance
	0	50	200	500	SEM	
Growth performance						
ADFI (kg)	0.08	0.08	0.07	0.08	0.001	NS
ADG (kg)	0.03^a^	0.03^a^	0.01^b^	0.01^b^	0.001	*
G:F ratio	0.38^b^	0.38^b^	0.14^a^	0.13^a^	0.014	*
Oxidative stress						
SOD (%)	45.2^C^	51.6^b^	60.3^ab^	69.7^a^	1.68	**
MDA (%)	32.5^a^	18.2^b^	11.0^c^	7.03^d^	0.36	**
CAT (μmoles/min/ml)	0.10	0.09	0.08	0.11	0.02	NS
Liver enzymes						
AST (units/L)	2.59^b^	2.89^b^	3.05^ab^	3.30^a^	0.11	*
ALT (units /L)	11.9^b^	39.4^a^	42.3^a^	44.3^a^	1.87	*

Values in the same row with different superscript letters differ (*P* < 0.05) *P < 0.05, **P < 0.01, *NS* not significant (*P* > 0.05)

*ADFI* average daily feed intake, *ADG* average daily gain, *G:F* gain to feed, *SOD* superoxide dismutase, *MDA* Malondialdehyde, *CAT* catalase, *AST* Aspartate aminotransferase, *ALT* alanine aminotransferase

### Interaction between stocking density and C. abbreviata extract on growth performance, oxidative stress and liver enzymes

**Fig. 1:**
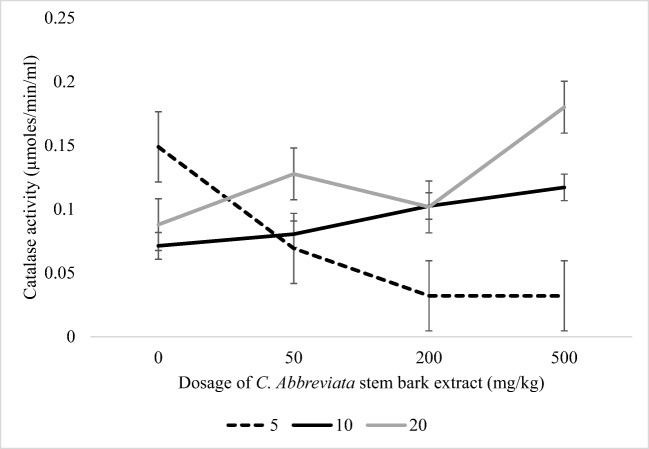
Interaction between levels of concentration of *C. abbreviata* extract and stocking density on catalase activity in indigenous chickens

There were no significant interactions (*P* > 0.05) between stocking density and dosage level of *C. abbreviata* extract on growth performance (Fig. 1). There was a significant interaction (*P* < 0.05) between the stocking density and dosage of *C. abbreviata* extract on CAT activity. A dosage of 500 mg/kg of *C. abbreviata* extract resulted to lower CAT in stocking density of 5 birds/m^2^ and higher CAT activity in stocking density of 20 birds/m^2^.

### Histopathological changes of liver of chickens administered with levels of C. abbreviata stem bark extract

**Fig. 2:**
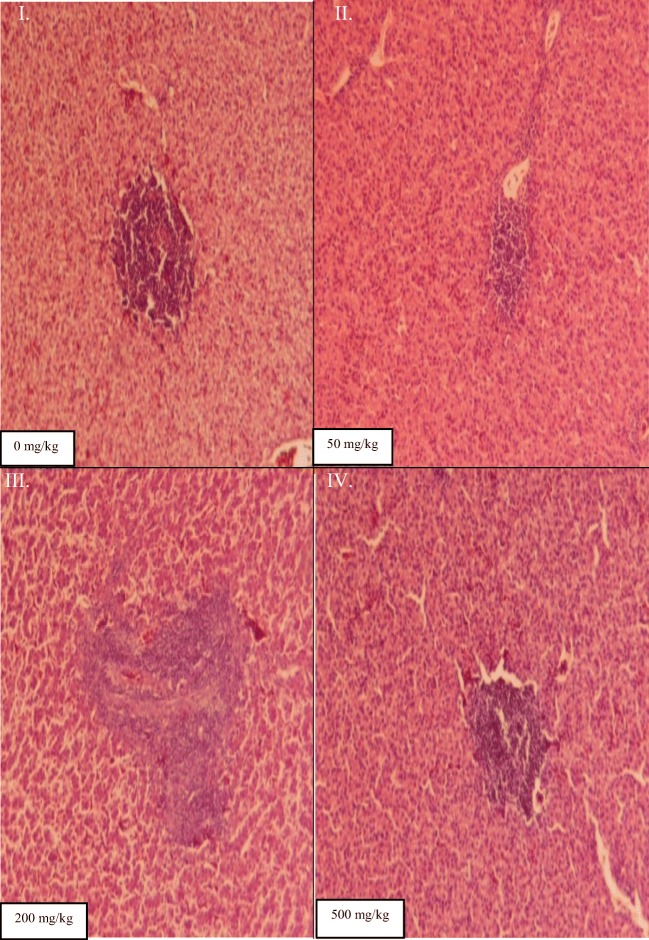
Photomicrographs represent cross-sections of histopathological changes of the liver of indigenous chickens administered with different dosages of *C. abbreviata* stem bark extract

There were no observed histopathological changes at different stocking densities of indigenous chickens. Nonetheless, marked changes were noted in the livers of chickens given different dosages of the *C. abbreviata* stem bark extract (Fig. 2). A normal liver sample of the group of chickens that did not receive a dose (0 mg/kg) of *C. abbreviata* extract had branching and anatomical cords radiating from the central vein with vesicular nuclei. Hepatocytes were within the normal limits with multifocal lymphocytic hepatitis and triaditis (Fig. 2: Image I). After administration of *C. abbreviata* extract with 50 mg/kg, liver exhibited mild congestions in the lymphocytic hepatitis. There were also necrotic changes observed in the liver. Hydropic swellings of hepatocytes were observed (Fig. 2: Image II). Similar observations were also noted in birds dosed with 200 mg/kg (Fig. 2: Image III). Following administration of 500 mg/kg of *C. abbreviata* stem bark extract, liver sample had few small focal areas of necropurulent hepatitis with scant accompanying epithelioid cells, macrophages and lymphocytes were observed (Fig. 2: Image IV).

## Discussion

The development of indigenous chicken as a commercial enterprise is contingent upon several factors including demand for welfare ethics. Crowding of chicken per given space is financial viable but compromises wellness (Dawkins, 2018). Therefore, it is crucial to determine stocking density standards for indigenous chicken to ensure its effective and welfare-friendly production. Findings that high stocking density (20 birds/m^2^) reduced ADFI correspond well with those of Tong et al. (2012) who reported a decrease in feed intake as stocking density increased in local chicken breed. In two separate studies, Simitzis et al. (2012) and Madilindi et al. (2018) reported a decrease in feed intake in birds kept in higher stocking density. Several studies have confirmed a decrease in feed intake as a result of overcrowding (Moreira et al., 2004; Thomas et al., 2004; Goo et al., 2018). Cengiz et al. (2015) reported that high stocking density distracts the mobility of birds in a given space. Thus, overcrowded birds battle to access feeders and drinkers. This may promote aggressive behaviour on birds such as pecking and threatening and reduce feeding behaviour. Male Ovambo chickens are normally aggressive (Joubert, 1996), which highlights the potential sensitivity to high stocking density. Increase in body temperature as a result of high stocking density may negatively influence feed intake. A higher ADG in stocking density of 5 birds/m^2^is similar to report by Mtileni et al. (2007) who cited that birds in lower stocking density were heavier than birds raised in high stocking density. These findings tantamount to the report by Tong et al. (2012) who reported a decline in ADG as stocking density rose in indigenous chickens. Dozier III et al. (2006) highlighted that increasing stocking density beyond 35 kg of BW/m^2^ suppressed final BW by 6%. Higher MDA levels in birds raised in high stocking density (20 birds/m^2^) agrees with findings of Simsek et al. (2009) who indicated that crowding enhanced oxidative destruction and caused MDA generation.

A growing body of evidence shows that most of stresses in poultry production, at the cellular level, are associated with oxidative stress (Surai, 2016). The use of extracts from medicinal plants in poultry to combat stress as a result of crowding is, therefore, vital and requires exploration. A lower ADG in birds administered with 200 and 500 mg/kg of stem bark extract of *C. abbreviata* compared with 0 and 50 ml/kg BW suggests that dosing birds with high concentration of *C. abbreviata* negatively affected growth performance. The presence of anti-nutritional bioactive compounds in *C. abbreviata*, even though unquantified, could be the reason of depressed ADG and gain to feed ratio in birds administered with 200 and 500 mg/kg. Sobeh et al. (2018) found that extract from root bark of *C. abbreviata* contain high content of phenolics such as proanthocyanidins (also known as condensed tannins). Condensed tannins bind with and engulf protein, compromising protein availability, digestibility and utilisation and this may have an indirect bearing on ADG (Huang et al., 2018). The presence of other phytochemicals such as alkaloids, steroids, terpenoids and saponins could have a detrimental effect on the performance of birds. It can be advised that stem bark extract from *C. abbreviata* should at most be dosed to 50 mg/kg, beyond this point, the growth performance is compromised.

Superoxide dismutase belongs to the first level of the antioxidant defence network (Surai, 2016). In addition, as important vitagene, it is the main driving force in cell or body adaptation to various stress conditions. High SOD in birds dosed with 200 and 500 ml/kg BW suggest that *C. abbreviata* stem bark extract was effective in enhancing SOD activity. This also reveals in vivo antioxidant properties of *C. abbreviata stem* bark extract. This is useful for birds because increase in antioxidant activity ensures proper and rapid elimination of reactive oxygen species that could be formed under high stocking density. Therefore, administrating birds with *C. abbreviata* stem bark extract could enhance scavenging of reactive oxygen species by increasing activity of SOD. Mongalo and Mafoko (2013) alluded that the extract of stem bark exhibited IC_50_ of 1.87 ± 0.25 mg/100 ml against 2,2-diphenyl-1-picrylhydrazyl (DPPH), which is a good scavenging characteristic of free radicals. In a separate but similar study, Wang et al. (2008) reported that *Forsythia suspensa* extract increased SOD levels in broiler chickens. Results that showed the lowest MDA levels in birds administered with 500 mg/kg of *C. abbreviata* dosage confirmed the antioxidant property of *C. abbreviata* stem bark extract. Lower levels of MDA indicate a reduction in oxidative damage (lipid peroxidation) as result of dosing birds with *C. abbreviata* stem bark extract.

The damage and recovery of liver is normally measured by the activity of serum transaminases such as AST and ALT (Atsafack et al., 2015). These enzymes are sensitive to toxic substances and play a crucial role in assessing pathological state of birds (Gudiso et al., 2019). Higher activity of AST and ALT birds dosed with 500 mg/kg of stem bark extract from *C abbreviata* concur with toxicological study by Atsafack et al. (2015) who reported an increase in AST and ALT in rats dosed with stem bark extract from *Schefflera barteri*. *Cassia abbreviata* stem bark extract contains phytochemicals that may have a propensity to increase the production of liver enzymes. Judging from high activity of AST and ALT activities, it is likely that stem bark extract from *C. abbreviata* has a hepatotoxicity effect when dosed at higher level such as 500 mg/kg. This is also verified by liver histopathological studies, which highlighted congestions in the lymphocytic hepatitis, necrotic changes and necropurulent hepatitis. Presence of phytochemicals such as tannins and alkaloids could have had negative impact on hepatica function.

An increase in CAT levels as a result of high stocking density conflicts with the results obtained by Simsek et al. (2009) which showed no change in serum CAT in broilers housed in high stocking density. The discrepancies could be due to different breed of chickens used and stocking densities. The activity of CAT is the defence that is responsible for converting hydrogen peroxide into water and oxygen (Surai, 2016). The removal of hydrogen peroxide protects cells against oxidative damage caused by hydrogen peroxide toxicity (Deepak et al., 2015). Higher CAT activity in birds kept under high stocking density when administered with 500 mg/kg of *C. abbreviata* stem bark extract suggests that this plant extract was involved in synthesis of CAT, an antioxidant enzyme responsible for adaptation of birds to oxidative stress.

## Conclusion

Increasing stocking density depressed growth performance and induced oxidative stress. Stocking density did not affect liver function. Surprisingly, high dose of *C. abbreviata* extract reduced growth performance. Dosage levels of *C. abbreviata* extract reduced oxidative stress. Dosage levels of *C. abbreviata* induced hepatotoxicity in birds. This suggests the need to gauge dosage level of *C. abbreviata* extract that will improve growth rate, minimize oxidative stress and be hepatotoxic-free in indigenous chickens.
